# Training in retinoscopy: learning curves using a standardized method

**DOI:** 10.1186/s12909-023-04750-y

**Published:** 2023-11-16

**Authors:** Antonio M. Estay, Iván Plaza-Rosales, Hernán R. Torres, Fabiola I. Cerfogli

**Affiliations:** 1 Department of Medical Technology, Faculty of Medicine, Universidad de Chile, Independencia #1027, 8380492 Santiago, Chile; 2 Department of Neuroscience, Faculty of Medicine, Universidad de Chile, Santiago, Chile; 3 Biomedical Neuroscience Institute, Faculty of Medicine, Universidad de Chile, Santiago, Chile

**Keywords:** Retinoscopy, Optometry, Learning curve, Standardization, Refractive errors, Teaching, Simulation training

## Abstract

**Background:**

Retinoscopy is one of the most effective objective techniques for evaluating refractive status, especially in non-cooperative patients. However, it presents a slow learning curve that often leads to student frustration. With the current Covid-19 pandemic and the need for social isolation, clinical education based on simulation has become more relevant. Therefore, we implemented retinoscopy laboratories and learning protocols to reduce student stress and learning time.

**Methods:**

We conducted a study to evaluate the retinoscopy learning curve using a new training protocol proposal. One hundred trainees were assessed in four stages, corresponding to 08, 12, 16, and 20 hours of training. Six different refractive defects were used trying to reproduce frequent conditions of care. The time spent on the assessment was not considered as additional training time. To analyze the data, we used non-parametric statistics and linear regression to assess the variables associated with training time and performance rate.

**Results:**

The mean performance score at 08 hrs was 32.49% (±16.69 SD); at 12 hrs was 59.75% (±18.80 SD); at 16 hrs was 70.83% (±18.53 SD) and at 20 hrs was 84.26% (±13.18 SD). Performance at 12 hrs was significative higher than 08 hrs of training, but did not show significant differences with the performance rate at 16 and 20 hrs. We found a strong positive correlation between performance and training time in retinoscopy (R = 0.9773, CI: 0.2678 - 0.9995 *p* = 0.0227).

**Conclusion:**

This study showed that an increasing number of hours of practice positively correlates with performance in retinoscopy. The elaboration of a protocol and standardization of performance per hour also allowed us to estimate that a minimum of 13.4 hrs of practice is required to achieve 60% performance. Using the resulting formula, it is possible to determine the number of hours of retinoscopy practice are necessary to reach a certain level of performance.

## Background

Spectacles prescription involves the detection, treatment, and follow-up of refractive errors, delivering graduation as the final product [1]. For this purpose, different diagnostic techniques are available, which can be divided into subjective and objective methods [2]. One of the most accurately objective refraction techniques is retinoscopy [3, 4]. It is a procedure that can be used in virtually all patients but is even more valuable in subjects with insufficient cooperation for subjective assessment [5, 6].

Even though retinoscopes were created before 1900, the first slit retinoscopes appeared around 1930 [7]. At present, a significant number of eye care professionals continue to have difficulties with the execution of this technique. The main challenge of this procedure is its slow learning curve to acquire the necessary expertise and the need for constant practice to maintain adequate levels of accuracy and proficiency. To accelerate this learning process and reduce the stress level of both patients and trainees as they develop this competency, the Department of Medical Technology from the University of Chile implemented a Retinoscopy and Refraction Laboratory in 2010. In addition to the laboratory, we developed a learning protocol to guide the trainees' training. The protocol has been improved from 2010 up to 2018 according to observation and currently available evidence. The final version was refined in 2018 and is the model used in this study (see *Methods* section). In the Chilean healthcare context, Medical Technologist in Ophthalmology and Optometry have been legally authorized to prescribe eyeglasses since 2011. Until that year, most teaching programs did not include retinoscopy in their academic curriculum. As a result, many professionals did not receive formal training in this area.

Although successfully performing retinoscopy requires a strategic pathway based on sub-goals [8], no studies have determined the learning curve to determine how much time trainees need to learn the retinoscopy technique. Educational programs are currently designed with a duration based on personal reports and experience. The present study aims to build a simulation station with a suitable clinical examination protocol to determine the time needed to reach an expected performance rate.

## Methods

### Study design

A retrospective study has been designed, using the results of performance rates obtained from different cohorts of trainees who spent training time in the Retinoscopy Simulation Laboratory between January 2019 and December 2022.

### Participants

The present study was approved by the Comité de Ética de Investigación en Seres Humanos (CEISH) at the Medicine Faculty of the Universidad de Chile. The study was conducted in strict accordance with the requirements of the Declaration of Helsinki. Both confidentiality and anonymity were guaranteed for all participants. To assess how much time trainees need to learn to perform retinoscopy correctly, we used the results of the performance rate obtained from different cohorts of trainees who practiced in the Retinoscopy Simulation Laboratory from January 2019 to December 2022. A total of 100 participants has been considered in the training process during this period. They were evaluated after 08, 12, 16, and 20 hrs of training. The time spent on the assessment was not considered as additional training time. All participants post-recruitment were assigned to one group, an undergraduate group (UG group), and a graduate group (G group). The UG group consisted of fourth-year Medical Technology trainees enrolled in the Ophthalmology and Optometry specialization, and the G group consisted of qualified Medical Technologists in Ophthalmology and Optometry professionals. Graduates have clinical experience but not specifically in the technique of retinoscopy.

### Procedures

We used the Heine Retinoscope Trainer (model 13301, manufactured in Germany) for learning the retinoscopy. This instrument has a range of simulated refractive errors of -7 to +6 spherical diopters. The front lens holder was used to include astigmatic defects using cylindrical lenses, recognizing their high prevalence in patients. Every participant used a Welch Allyn Retinoscope (model 18200). The principal elements of the learning protocol is based in sub-goals [8], and the used were:

1. Standard Table and Chair Height: The utilization of standard table and chair dimensions is implemented to minimize the likelihood of trainees resting their arms on the table.
2. Retinoscope Configuration: The retinoscope is set to the plane mirror position, and a distance correction lens is added to reduce errors associated with these parameters.
3. Heine Retinoscope Trainer: Every student uses the Heine Retinoscope Trainer to eliminate bias arising from external factors [9]. This trainer has a high accuracy, precision, and visual axis indicator [10].
4. Individual Working Distance and Compensation Lens: Each student's working distance and compensation lens are calculated individually, maximizing the distance and continuously monitoring it during training.
5. Constant Body Posture Correction: Emphasis is placed on correcting body posture consistently, particularly during the initial four hours of training. A variation of just 5,58 degrees of visual axis is associated with a significative lens power error (this has been reviewed in myopes and astigmatic patients) [11].
6. Sequential Training Phases: The first four hours focus exclusively on training for spherical errors, aiming to achieve an error margin of less than 0.25 diopters. After the fifth hour, training for sphero-cylindrical defects commences, with a progressive reduction of pupil size from 8 mm to 2 mm.
7. Performance Assessment: Performance tests are conducted at 08th, 12th, 16th and 20th hours of retinoscopy training. Time used in testing is not considered as part of the training time.
8. Pupil Size: During the execution of retinoscopy, trainees frequently encounter a diverse range of pupil responses and sizes because this procedure is often conducted in scotopic (low light) conditions, utilizing the retinoscope for illumination. This phenomenon has been investigated in prior studies, with Cardona et al. providing insights into pupil size dynamics. They reported that the mean pupil size in mesopic (medium light) conditions averages 5.4 mm (±0.6 mm), while in photopic (high light) conditions, it measures 2.3 mm (±0.5 mm) [12]. Similarly, Chen et al. offered valuable findings, indicating that under photopic illumination, the mean pupil diameter was 4.26 mm (±0.95, SD) within a range of 2.08 to 7.13 mm. Under scotopic illumination, the mean pupil diameter measured 6.09 mm (±1.00, SD) between 3.43 to 8.13 mm [13]. To address this variability, six retinoscopy trainers are employed during evaluation, each configured to simulate a different spherocylindrical defect. Trainees have up to six minutes to resolve each defect before rotating to the next station. The key features of the simulated defects at each station encompass:
  - Two low-difficult defects (mild refractive error, pupil size between 6 – 8 mm).
  - Two moderate-difficult defects (moderate refractive error, pupil size between 4 – 5 mm).
  - Two high-difficult defects (one high refractive error with 6 mm pupil size, and one mild refractive error, but with a 2mm pupil size).

The error tolerance range used to consider a student's response as correct is set at +/- 0.25 diopters for mild and moderate defects and +/- 0.5 diopters for high defects [14, 15]. The range of error is calculated considering the sum of the difference of spherical and cylindrical error.

### Statistical analysis

Data collection and statistical analyses were performed using Microsoft Excel 365 (Microsoft Corporation, Redmond, WA, USA) and GraphPad Prism version 8.0.1 for Windows 10 (GraphPad Software, San Diego, CA, USA). Descriptive statistics were described as frequencies and percentages, and continuous variables as mean and standard deviation (SD). The normal distribution of the parameters was assessed using the Shapiro-Wilk normality test. The Mann-Whitney U test was performed to compare the mean performance rate between the first two sections of retinoscopy practice time (08 and 12 hrs). Furthermore, for comparison of more than two groups, Kruskal-Wallis test was used, corrected by Dunn’s multiple comparisons tests. Pearson’s correlation and Linear regression approaches with their 95% confidence interval were used to assess the correlation and the best adjustment between the performance rate in percentage (%) and the retinoscopy practice time (08, 12, 16, and 20 hrs). A *p*- value < 0.05 was considered statistically significant.

## Results

A total of 100 subjects was included in this study (34 men and 66 women). 36 subjects represented UG trainees (17 were men [47,22%], and 19 were women [52,77%]), and 64 subjects represented G trainees (17 were men [26,56%], and 47 were women [73,44%]).

### Frequency distribution of performance scores

**Fig. 1:**
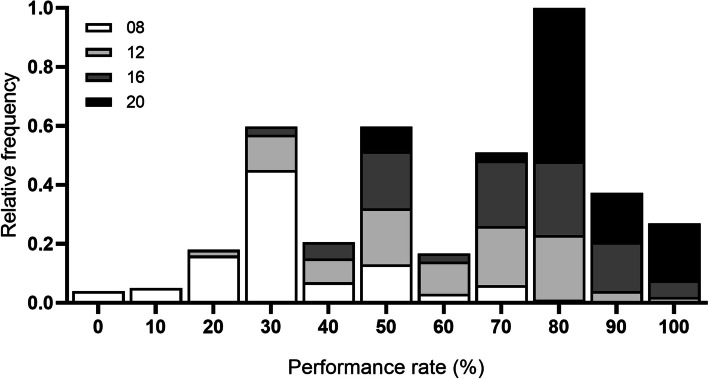
Frequency distribution of the performance rate to different retinoscopy practice times. Was evaluated the performance of the group at 08, 12, 16 and 20 hrs. The histogram shows the data as the relative frequency of the percentage of success by participants

The data showed a non-normal distribution (Shapiro-Wilk normality test, *p* > 0.05). We obtained the following specific normality test for each retinoscopy practice time (Shapiro-Wilk test; 8 hrs: W = 0.9557, *p* = 0.002; 12 hrs: W = 0.9741, *p* = 0.046; 16 hrs: W = 0.9278, *p* = 0.021 and 20 hrs: W = 0.8053, *p* < 0.0001, respectively). The frequency distribution of performance rate was inspected for each retinoscopy time, as shown in Fig. 1 and Table 1, where the mean and SD at 08 hrs (*N =* 100) was 32.49% (±16.69 SD), at 12 hrs (*N =* 100) was 59.75% (±18.80 SD), at 16 hrs (*N =* 36) was 70.83% (±18.53 SD) and at 20 hrs (*N =* 36) was 84.26% (±13.18 SD), respectively.

**Table 1:** Retinoscopy performance data as a function of training hours

**Training (hr.)**	**Mean**	**SD**	**LL-UL (95% CI)**	**Sample (n)**
**8**	**32.49**	**16.69**	**29.19 – 35.81**	**100**
	UG 28.01	16.08		36
	G 35.03	16.61		64
**12**	**59.75**	**18.80**	**56.02 – 63.48**	**100**
	UG 47.45	16.76		36
	G 66.67	16.27		64
**16**	**70.83**	**18.53**	**64.56 – 77.10**	**36**
**20**	**84.26**	**13.18**	**79.80 – 88.72**	**36**

*Hr* hours, *SD* Standard Deviation, *LL* Lower Limit, *UL* Upper Limit, *CI* Confident Interval, *n* number, *UG* Undergraduate, *G* Graduate

### Analysis of performance by hours of practice

**Fig. 2:**
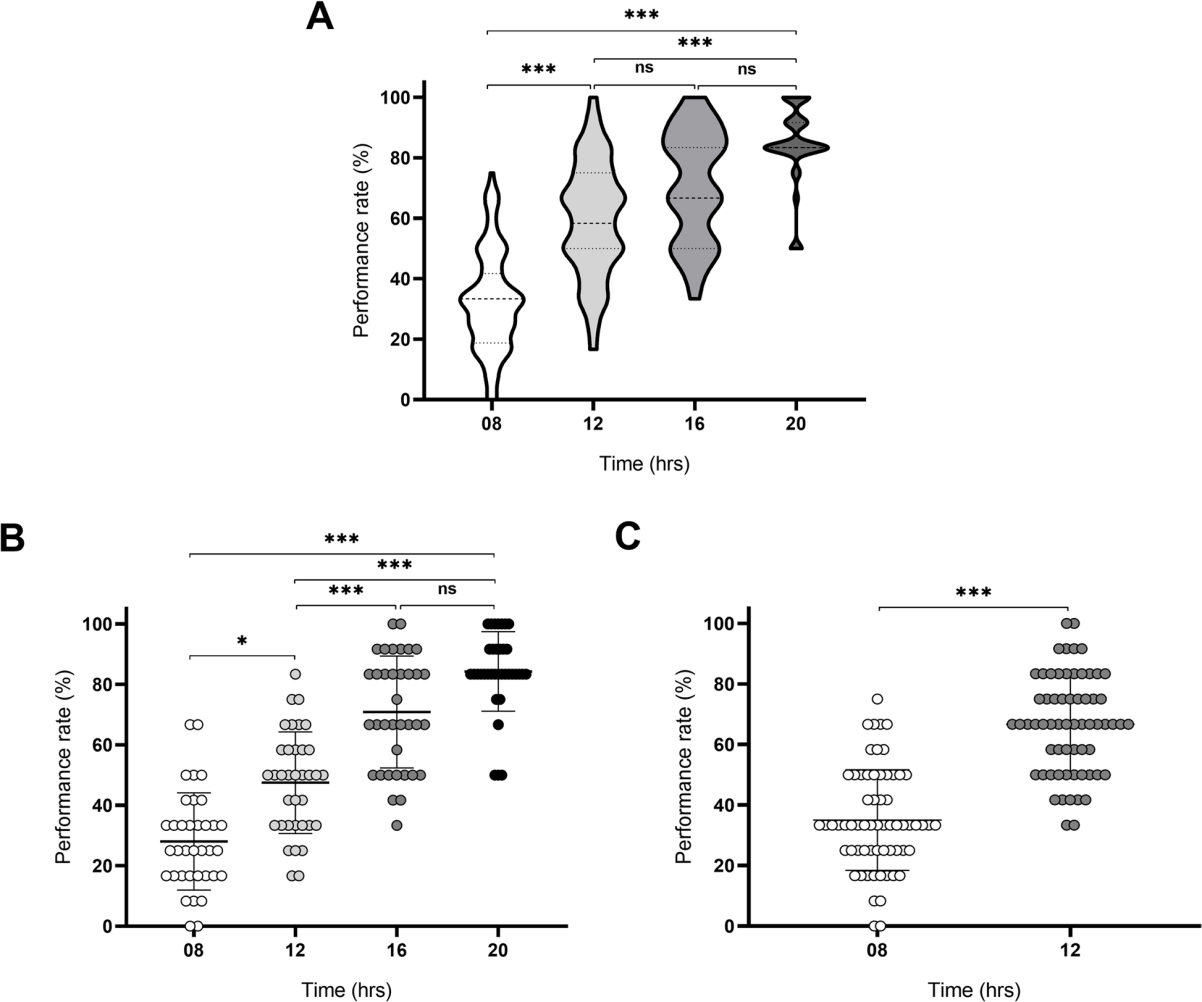
Effects of retinoscopy practice time on the performance rate. **A** Analysis for differences in the complete group, **B** Analysis for differences in the Undergraduate group, **C** Analysis for differences in the Graduate group. For each time (08, 12, 16 and 20 hrs). Data are presented as mean and (± SD), using Mann-Whitney U test or Kruskal-Wallis test. A *p*-value less than 0.05 was considered statistically significant. Significance is indicated by the following symbols: * *p* < 0.05, ** *p* < 0.01, *** *p* < 0.001, **** *p* < 0.00001, ns = not significant. Post hoc analyses were applied (Dunn's test, *p* < 0.05)

A Kruskal-Wallis test was conducted to determine the effect of different retinoscopy practice times on performance. We assumed an unpaired experimental design and a non-Gaussian data distribution. The average rate of return at times of practice was significantly different (H = 145.9, *p* < 0.0001). Post hoc analyses, adjusted for multiple comparisons (Dunn's multiple comparisons tests), revealed that the performances between 08 hrs vs. 12 hrs, 16 hrs vs. 20 hrs, as well as more than 12 hrs vs. 20 hrs were significantly different (*p* < 0.0001). Meanwhile, Fig. 2 shows that the performance did not differ significantly between 12 hrs vs. 16 hrs as well as 16 hrs vs. 20 hrs (*p* = 0.1383 and *p* = 0.1767, respectively). To study whether there are effects related to the basal practice of the trainees, we separated the sample into groups consisting of UG and G trainees. Given that the UG group (*N =* 36) completed four times of retinoscopy training (08 hrs, 12 hrs, 16 hrs, and 20 hrs). The results of Fig. 2B show significant differences for all comparisons (except of 16 hrs vs. 20 hrs; *p* = 0.1924), using the Dunn’s multiple comparison test. On the other hand, the G group (*N =* 64) only completed the first two sections of retinoscopy practice times. We found that the mean performance rate between 08 and 12 hrs was significantly different (Mann-Whitney U test, *p*< 0.0001), as shown in Fig. 2C.

### Performance in undergraduate and graduate trainees

**Fig. 3:**
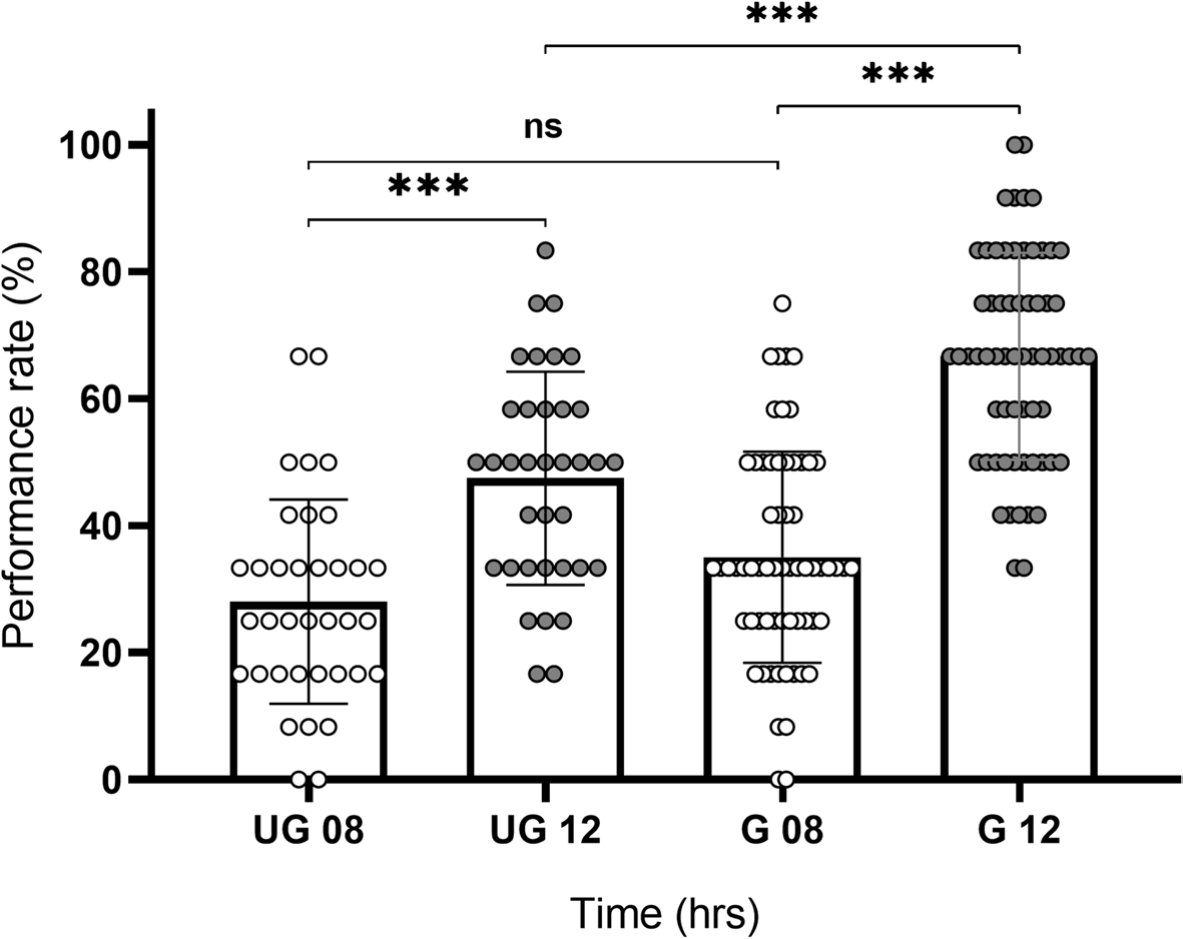
Comparison of performance in undergraduate and graduate trainees. The data shows an analysis of the performance rate at 08 and 12 hrs between UG and G groups. Data are presented as mean and (± SD), compared using Kruskal-Wallis test. A *p*-value less than 0.05 was considered statistically significant. Significance is indicated by the following symbols: * *p* < 0.05, ** *p* < 0.01, *** *p* < 0.001, **** *p* < 0.00001, ns = not significant. Post hoc analyses were applied (Dunn's test, *p* < 0.05)

Having established that both the UG and G groups could improve their performance during the practicing time, we decided to determine the differences between the groups for the same practice time. The results in Fig. 3 show that the performance rate is significantly different between both groups (Kruskal-Wallis test; H = 94.05, *p* < 0.0001). Using the Dunn's multiple comparisons test, significant differences were detected for UG12 vs. G12 (*p* = 0.0005) but not for UG08 vs. G08 (*p* = 0.7023). These results suggest that the effect of better performance may be influenced by the trainee's previous experience and time in the clinic, following 12 hrs of standardized retinoscopy training.

### Regression analyses

**Fig. 4:**
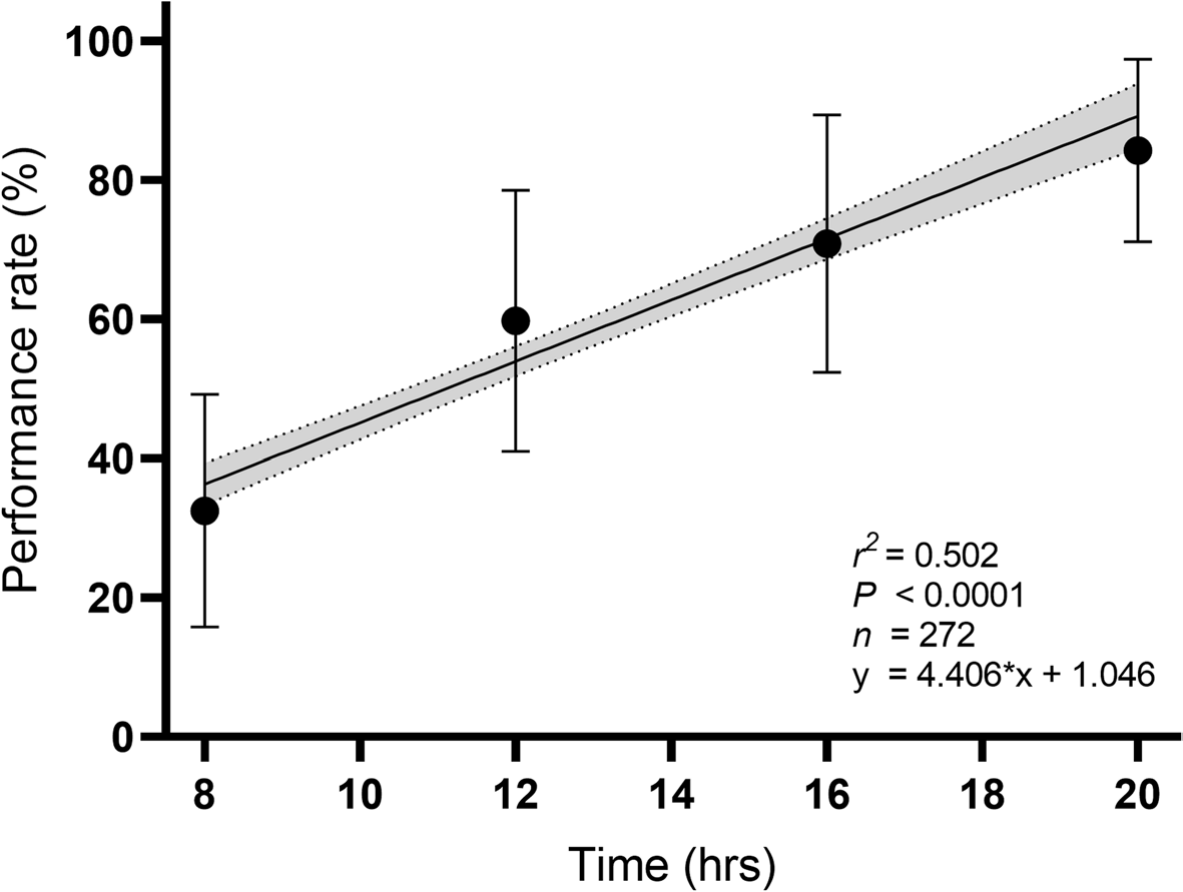
Linear Regression of performance rate as a function of retinoscopy practice time. The data were strongly adjustable to linear regression (R^2^ = 0.5015, *p* < 0.0001). The graph shows the average value for each training time

We found that the performance rate and retinoscopy practice time showed a statistically significant positive correlation (Pearson's correlation *r* = 0.9773, *p* = 0.0227, 95% CI = 0.2678 - 0.9995). We computed the result from the means of each practice time. Additionally, we performed a linear regression to assess the relationship between retinoscopy practice time and performance, representing the explanatory variables and dependent variables, respectively. As shown in Fig. 4, the data were strongly adjustable to linear learning of the retinoscopy technique according to the practice time (*R*^*2*^ = 0.5015, *p* < 0.0001). Considering the performance rate, data obtained formula has been achieved. This equation allows to predict the student's performance related to the training time:

1 $${\varvec{y}}\boldsymbol{ }=4.406\boldsymbol{*}{\varvec{x}}+\boldsymbol{ }1.046$$

## Discussion

The objective of this study was to evaluate the Retinoscopy learning curve using a new training protocol proposal. Although numerous studies have explored methods for evaluating the refractive error and retinoscopy is an evaluation technique used for more than 100 years [7, 16], almost no information has been published about the learning process of this procedure. This study demonstrates for the first time that using a standardized method of learning retinoscopy permits to predict the performance rate and establishes the minimum time needed to learn this challenging technique [17]. It is a belief in optometry that many years of practice are necessary to develop an acceptable level of performance in retinoscopy [8]. However, we described that the practice time of less than 14 hrs is sufficient to reach an 60% performance rate.

The same is true for other skills in ophthalmology, in which mastering the ability is based on constant and persistent training during clinical practice [18]. Mainly due to several variables that the practitioner must control when applying this technique; many of them even tend to avoid its use [19, 20]. In recent decades, many changes have occurred in the manner of how ophthalmic techniques have been taught due to new technologies and educational requirements but particularly Medical Education worldwide was greatly affected by the Covid-19 pandemic, requiring a rapid adaptation of many educational programs [21]. Among these innovations and adaptations was the joint use of cellular technology with the retinoscopy technique, incorporating learning through web-based retinoscopy simulators [22]. Despite the efforts, digital tools and distance learning cannot fully replace face-to-face training in the technique [23].

In this study, we aimed to assess the learning curve for retinoscopy using a novel training protocol. We included 100 trainees at different stages of training, then evaluated their performance at 08, 12, 16, and 20 hours, and incorporated six refractive defects commonly encountered in clinical practice. Our intention was not to compare students with professionals but to observe how trainees' skills evolved. By including students and professionals in the same sample, we aimed to bolster the statistical robustness of our findings and demonstrate a consistent learning curve across the entire group, irrespective of their initial expertise level. This approach helped mitigate potential biases and strengthened the overall validity of our study. All trainees in the Graduates group participating in this study originated from various training programs and universities, exhibiting a spectrum of educational backgrounds. On the other hand, some individuals had not undergone formal retinoscopy training, while others possessed a higher training level in this skill. To mitigate potential bias stemming from prior exposure to the study protocol during their undergraduate education, excluded from the group of graduates were those who had received training using the protocol of the present study to obtain the title of Medical Technologist.

In the study, we proposed that standardization is essential to optimize resources and time to reduce inter-observer variability observed in other studies [24, 25]. Frequently, researchers report problems associated with different retinoscopy techniques [26]. Our study protocol was designed to focus on pupils with a diameter between 4 and 8 mm, including a scenario with a 2-mm pupil. Specifically encompasses the following: two cases of low-difficulty defects, characterized by mild refractive abnormalities and pupils between 6 and 8 mm in diameter; two cases of moderate difficulty, involving moderate refractive irregularities and pupils between 4 and 5 mm; and finally, two scenarios of high-difficulty, comprising a high refractive defect accompanied by a 6-mm pupil, as well as a mild refractive defect coinciding with a 2-mm pupil. To reduce variability, it is necessary to consider as a precedent that during the development of the retinoscopy protocol, it is essential to control the body posture and working distance during the first four hours to improve precision. Studies also established that those two elements are considered the principal source of error in retinoscopy [11, 20]. A clear example is the variability detected between experienced teachers in retinoscopy that has been ±0,87 D and between trainees ±3,15 D [27]. When correcting for high ametropia, the variability is even higher. Teaching retinoscopy through a simulator or trainer considerably reduces that error. At the same time, the assessment of the retinoscopy learning curve should be performed by applying an optimized protocol using a retinoscopy trainer. Furthermore, when we eliminate interobserver variability, trainees that use this learning process have greater accuracy with this technique and retinoscopy can be a better starting point for subjective refraction instead of the autorefractor [5, 7, 24]. This comprehensive approach ensures a thorough investigation of retinoscopy across a spectrum of pupil sizes and other complexities, contributing to the robustness and applicability of our study results.

A detailed examination of each trainee's individual performance at different levels of difficulty is beyond the scope of our current research. The data set available to us mainly comprises the performance rates achieved by each trainee after 8, 12, 16, and 20 hours of training. However, one noteworthy observation has emerged, indicating that trainees face higher failure rates when faced with cases of greater complexity, characterized by more severe refractive defects or smaller pupil sizes. Unfortunately, we do not currently have the specific data. Nevertheless, it is essential to emphasize that our study was designed to measure the learning curve under conditions that closely mirrored real-world clinical scenarios, thus increasing its ecological validity, and at this stage of the project add more high-difficult defects might have contradicted the main goals of our learning protocol. Its aims ranged from skill acquisition, optimization of learning efficiency, reduction of total learning time, and mitigation of the inherent stress associated with mastering this technique. Balancing these objectives was essential to maintaining the ethical and pedagogical integrity of the study. It should be noted, after finishing the training with high defects and small pupil sizes, trainees say it was easier to carry out retinoscopy in their clinical practice with patients. This fact is an important issue to show retinoscopy as a skill that requires meta-cognitive strategies for the integration of declarative knowledge addition to many procedural skills, for its total acquisition. For future research, we recommend the inclusion of outcomes categorized by degree of difficulty adding more high-difficult defects. This methodology would provide a more nuanced understanding of how learners' proficiency evolves in response to different levels of challenge in the retinoscopy domain. Such an exploration would provide valuable information on the learning curve associated with this skill and could inform specific training strategies to improve trainee performance across a broad spectrum of clinical settings. This approach has the potential to offer supplementary insights into the adaptability of trainees when confronted with challenging clinical scenarios [8].

This study shows evidence that the skills necessary to perform retinoscopy to an acceptable level (60% accuracy) can be achieved during a period of training of a minimum of 13,4 hrs, but also show that the progress of the learning curve is linear until 20 hrs of training under the condition of using the proper protocol. These results allow us to establish a minimum standard training practice to learn retinoscopy. Furthermore, it was possible to see differences in the performance score and learning curve in both groups. The UG group achieved 12 hrs of training with a median of 50% of the performance rate, whereas the G group achieved at the same time (12 hrs) a median performance rate of 66,7%. These results are consistent with other studies proposing an added benefit to optometry education to incorporate tools to measure the optometrist's diagnostic reasoning and show differences between trainees and professionals with at least three years of experience [28]. Based on the evidence, we confirmed the importance of training time and previous experience in better performance of the retinoscopy technique.

Finally, considering the characteristics of our sample, we can recognize differences within the graduate group that can attributed to both prior clinical experience and undergraduate education. It is relevant to acknowledge that this aspect represents a specific limitation of our study. Our limitation arises from the inherent uncertainty regarding whether the fundamental components of retinoscopy training were integrated into their respective educational programs. In addition, it should be noted that the number of years since graduation of students in this group may influence the observed results. As such, future investigations might explore two potential research avenues. First, an analysis could be performed to assess the level of complexity of retinoscopy considering variations in pupil size and high defects. Concurrently, efforts could be directed towards comparing the performance of undergraduate and graduate students, shedding light on potential differences in their skill development and aptitude in this domain. On the other hand, the final goal would be to evaluate performance in a clinical setting and with patients. These guidelines would contribute to a complete understanding of retinoscopy training and competency.

## Conclusion

The present study demonstrated a consistency between retinoscopy performance rate and training time. A learning curve has been established based on the obtained results. The elaboration of a protocol and standardization of performance per hour also allowed us to estimate that a minimum of 13.4 hrs of practice is required to achieve 60% performance. According to these findings, it is possible to conclude that retinoscopy is a skill that requires meta-cognitive strategies for the integration of declarative knowledge in addition to procedural skills, where it is necessary for training time to improve performance.

## Data Availability

The datasets used and/or analyzed during the current study available from the corresponding author on reasonable request.

## Abbreviations

Covid-19: Coronavirus disease
UG group: Undergraduate group
G group: Graduate group
± SD: ± Standard Deviation

## References

[1] Kaur K, Gurnani B: Subjective Refraction Techniques. In: StatPearls. edn. Treasure Island (FL): StatPearls Publishing 2022. 35593807

[2] Rodriguez-Lopez V, Dorronsoro C (2022). Beyond traditional subjective refraction. Curr Opin Ophthalmol.

[3] Asiedu K, Kyei S, Ampiah EE (2016). Autorefraction, Retinoscopy, Javal's Rule, and Grosvenor's Modified Javal's Rule: The Best Predictor of Refractive Astigmatism. J Ophthalmol.

[4] Akil H, Keskin S, Çavdarli C (2015). Comparison of the refractive measurements with hand-held autorefractometer, table-mounted autorefractometer and cycloplegic retinoscopy in children. Korean J Ophthalmol.

[5] Jorge J, Queiros A, Gonzalez-Meijome J, Fernandes P, Almeida JB, Parafita MA (2005). The influence of cycloplegia in objective refraction. Ophthalmic Physiol Opt.

[6] Wallace DK, Carlin DS, Wright JD (2006). Evaluation of the accuracy of estimation retinoscopy. J AAPOS.

[7] Fau C, Nabzo S (2018). Copeland streak retinoscope. Arch Soc Esp Oftalmol (Engl Ed).

[8] Hollis J, Allen PM, Heywood J (2022). Learning retinoscopy: A journey through problem space. Ophthalmic Physiol Opt.

[9] Rotsos T, Grigoriou D, Kokkolaki A, Manios N (2009). A comparison of manifest refractions, cycloplegic refractions and retinoscopy on the RMA-3000 autorefractometer in children aged 3 to 15 years. Clin Ophthalmol.

[10] HEINE Skia/Retinoscope Trainer Instructions. Gilching: HEINE Optotechnik GmbH & Co. KG; 2020.

[11] Tay E, Mengher L, Lin XY, Ferguson V. The impact of off the visual axis retinoscopy on objective central refractive measurement in adult clinical practice: A prospective, randomized clinical study. Eye (Lond). 2011;25(7):888–92. 10.1038/eye.2011.79 PMC3178162 21494285

[12] Cardona G, López S (2016). Pupil diameter, working distance and illumination during habitual tasks. Implications for simultaneous vision contact lenses for presbyopia. J Optometry.

[13] Chen L, Chernyak D (2013). Pupil Changes under Scotopic and Photopic illumination. Investig Ophthalmol Vis Sci.

[14] Goss DA, Grosvenor T (1996). Reliability of refraction–a literature review. J Am Opt Assoc.

[15] Smith G (2006). Refraction and visual acuity measurements: what are their measurement uncertainties?. Clin Exp Optometry.

[16] Thorington J (1897). Retinoscopy (or shadow test) in the determination of refraction at one meter distance, with the plane mirror.

[17] Lee O: Retinoscopy 101. https://www.aao.org/young-ophthalmologists/yo-info/article/retinoscopy-101. (2015) Accessed 02-28-2023.

[18] Donovan L, Brian G, du Toit R (2008). A device to aid the teaching of retinoscopy in low-resource countries. Br J Ophthalmol.

[19] Kenneth Campbell E (1903). The Theory of Retinoscopy. Lancet.

[20] Buser A. Objektive Refraktionsbestimmung - Skiaskopie. Klin Monbl Augenheilkd. 2014;231(8):841–55. 10.1055/s-0033-1357960 25133562

[21] Rajhans V, Memon U, Patil V, Goyal A (2020). Impact of COVID-19 on academic activities and way forward in Indian Optometry. J Optom.

[22] Chandrakanth P, Gosalia H, Verghese S, Narendran K, Narendran V (2022). The Gimbalscope - A novel smartphone-assisted retinoscopy technique. Indian J Ophthalmol.

[23] Acosta ML, Sisley A, Ross J, Brailsford I, Bhargava A, Jacobs R, Anstice N (2018). Student acceptance of e-learning methods in the laboratory class in Optometry. PLoS One.

[24] Jorge J, Queirós A, Almeida JB, Parafita MA. Retinoscopy/Autorefraction: Which is the best starting point for a noncycloplegic refraction? Optom Vis Sci. 2005;82(1):64–8. 15630406

[25] Acuña L, Uribe MM, Orozco LC. Evaluación de la reproducibilidad de la retinoscopia dinámica monocular de Merchán. Colombia Médica. 2009;40(4):399–407.

[26] Saunders KJ, Westall CA (1992). Comparison between near retinoscopy and cycloplegic retinoscopy in the refraction of infants and children. Opt Vis Sci.

[27] García Lozada D. Concordancia interobservadores en retinoscopía estática entre docentes y estudiantes de optometría de una institución universitaria de Bogotá, Colombia. Investigaciones Andina. 2011;13(23):285–92.

[28] Edgar AK, Ainge L, Backhouse S, Armitage JA (2022). A cohort study for the development and validation of a reflective inventory to quantify diagnostic reasoning skills in optometry practice. BMC Med Educ.

